# Orange palpebral spots: A case presentation

**DOI:** 10.1177/2050313X221082435

**Published:** 2022-03-05

**Authors:** Mahaveer S Sangha, Hazem AH Ibrahim, Rhonda Meys

**Affiliations:** 1 University College London Medical School, London, UK; 2 Department of Dermatology, Royal Free London NHS Foundation Trust, London, UK

**Keywords:** Orange, orange palpebral spots, orange palpebral macules, periocular, lipofuscin, sun damage, solar elastosis

## Abstract

Orange palpebral spots are described as bilateral, ovoid, poorly defined orange-yellow macules on the superior eyelid and are predominantly reported in Caucasian populations. Previous reports have found correlations with melatonin incontinence secondary to trauma, lipofuscin accumulation in patients with superficial fatty tissue and palpebral thinness, and vitamin E, carotenoid and beta-cryptoxanthin levels. We present, to our knowledge, the first case of orange palpebral spots reported in the United Kingdom, in a patient with a background of atopy, significant sun exposure, bilateral cataracts and retinal detachment. The 59-year-old male initially presented with a dorsal nasal lesion with the differential: basal cell/trichoblastic carcinoma. During his excisional Mohs surgery, bilateral orange-yellow discolourations of the superior palpebrae were noted. The history was not significant for consumption of dietary sources of pigmentation, such as carotenoids, xanthophylls and vitamin E – found in green leafy vegetables and nut oils, respectively. The age of onset was unknown. A diagnostic skin punch biopsy was suggestive of orange palpebral spots and showed thinning of the epidermis, high-situated superficial and mature fat cells, with minimal pigment incontinence and perivascular lymphocytic infiltration. In addition, solar elastoses were identified on histology. After review in our local clinic-pathological meeting and of the published literature, a diagnosis of orange palpebral spots was given. The pathogenesis of orange palpebral spots remains to be elucidated. The role of sun exposure as a contributing factor to the generation of orange palpebral spots is therefore discussed.

## Introduction

Orange palpebral spots (OPS), also described as orange palpebral macules or patches, were first reported by Assouly et al.^1^ in 2008. OPS present as bilateral, ovoid, poorly defined orange-yellow macules on the superior eyelid. Orange discolouration is more pronounced near the medial canthus with yellow hues towards the mid-superior eyelid. Pathogenesis is still unknown, previous studies posit deposition and accumulation of dietary elements in the reticular and superficial dermis or metabolic/endocrine processes to be possible causes.

OPS differs from xanthoma/xanthelasma by lesion appearance (bilateral and symmetrical distribution), and microscopically. Histologically, OPS presents with normal adipocytes/adipose tissue located high in the dermis, contrasting with lipid-laden macrophages seen in xanthelasma. Likewise carotenoderma, necrobiotic xanthogranuloma and toxin/medication-related discolouration can be clinically excluded through examination, history and investigation. Biochemically, reports suggest OPS has weak association with raised vitamin E, carotenoid and beta-cryptoxanthin levels.^1^ No relation to lipid panel results have been documented.

To our knowledge, there have been five previous publications reporting patients with OPS. Including this case report, information from 35 individuals in total has been recorded (27 females and 8 males, 34 from previous publications). Studies suggest true prevalence may be greater as this disease often goes unnoticed by individuals and physicians.^1–5^ The average age of presentation is 54 years (range 28–79 years). Time since onset was commonly unknown and all patients were of Caucasian ethnicity either from Europe (UK, France) or North America (USA, Canada). Thirteen patients featured typical aspects of OPS on histology – superficially situated adipose tissue/cells. In the remaining 22 cases the characteristic pathology was either absent (one) or unrecorded (21). Medical histories were not significant for metabolic/endocrine disease or hyperlipidaemia. One patient had a history of atopy, one patient had elevated levels of vitamin E and five had elevated levels of the beta-cryptoxanthin carotenoid. Two patients (inclusive of this case report) had a prior history of ocular disease including cataracts (two) and age-related macular degeneration (one). Self-report of excessive intake of fruit and vegetables was recorded in 10 patients, with the remaining reporting a balanced diet, normal intake or remaining unassessed. No correlation with medication or compounds present in blood has been reported.

## Case

**Image 1.**
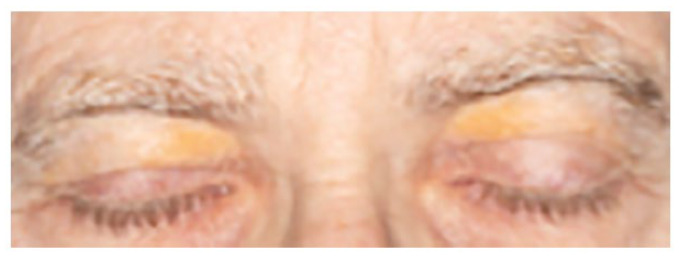
Bilateral and symmetrical orange-yellow patches with greatest colour intensity medially and lightening on approach to the middle superior palpebrae – characteristic of OPS.

**Figure 1.**
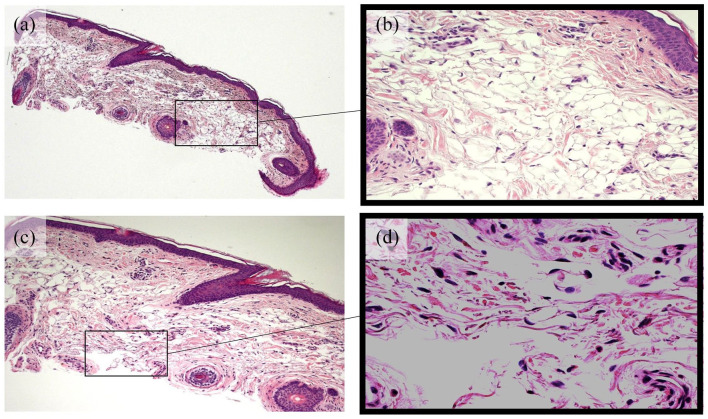
Characteristic fat in the upper dermis seen in OPS at (a) 5× magnification and (b) 20× magnification and solar elastosis from sustained sun exposure at (c) 10× magnification and (d) 40× magnification seen in the same region of the biopsy. Haematoxylin and eosin.

In this case report, we present a 59-year-old male with type II skin who was referred due to a nasal bridge lesion (diagnosed histologically as nodular basal cell carcinoma) and was incidentally found to have yellow-orange gradient discolorations of the superior eyelid on examination (Image 1). The patient had a history of atopy and reported significant lifetime sun exposure (also evidenced by solar elastoses on histology – Figure 1), but no endocrine/metabolic disease history. Surgical history is significant for bilateral cataract extraction a decade prior to OPS identification and retinal detachment of both eyes. Histologically, a thin epidermis, high-situated superficial and mature fat cells (Figure 1), minimal pigment incontinence and perivascular lymphocytic infiltration were identified. This is the first formal report of a patient with OPS in the United Kingdom. In presenting this case we hope to contribute to the eventual clarification of the disease process and put forward our hypotheses on possible routes of pathogenesis.

## Discussion

Pigment and skin changes have been associated with autoimmune, metabolic or endocrine disease, for example, acanthosis nigricans, ‘bronzed’ hemochromatosis and vitiligo. Similar processes are unlikely to be responsible in view of the histology and general well-being of patients with OPS. Another explanation is age-related lipofuscin, a lysosome-associated pigment, accumulation. In patients with unusual anatomy of superficial fatty tissue and thin palpebral skin, the lipofuscin pigment-laden fat cells may cause the coloration in OPS.^1^ However, current evidence is limited to one biopsy from Assouly et al.’s study which was not compared with a control; furthermore, inconsistent staining techniques between studies make it difficult to ascertain the validity of this result. Moreover, OPS is not exclusive to the elderly. Other pigments, particularly those with dietary sources, are less likely as though some are lipophilic and may be stored/accumulated in fatty tissue, histological assessment has not been reproducible and correlations with blood concentrations have not been identified.

Belliveau et al.^2^ have suggested that melatonin incontinence secondary to trauma, such as eye rubbing, may be responsible for this pigmentation pattern. Chronic atopy-related eye rubbing was not reported in this case, however. Ophthalmic surgery was, however, reported. The patient reported cataract extraction and retinal detachment repair to both eyes. This could be significant as accumulation of metabolites is linked to retinal degeneration^6^ and may contribute to gradual weakening of adhesion between neuroepithelium and pigment epithelium, thus increasing the risk of retinal detachment. Lipofuscin could be one such metabolite in OPS, however, its presence in OPS is not widely reported and was not investigated for in our patient.

We have presented the first patient with self-reported increased sun exposure, which was objectively identified by solar elastoses on histology (Figure 1). OPS may present a variation in the spectrum of normality that is triggered in certain individuals due to palpebra thinning secondary to increased sun exposure. The eyelid’s skin is constitutionally thin and studies have shown that ultraviolet (UV) radiation results in further skin atrophy.^7^ Moreover, the medial aspect of the superior eyelid houses adipose tissue in the nasal/medial and pre-aponeurotic fat pads. This provides a potential reason for OPS’ predilection to the medial aspect and only rare lower or extensive upper lid involvement. Similarly, longstanding and sustained sun exposure/damage accelerates lipofuscin accumulation and may explain the mechanism by which hypothesised pigment accumulation can occur, superimposed on normal ageing.^8^

It is unfortunately not known whether previously reported patients also experienced increased levels of sun exposure. In addition, pigment deposition (e.g. palpebrae lipofuscin) variation should be established in a control population to identify whether thinning or pigmentation is primarily, if at all, responsible.

## Conclusion

The clinical importance of OPS still remains unknown. There is a possibility it presents as a marker for premature ageing or of greater susceptibility to sun damage.
